# Facilitating dental disease screening program in prisoners using an intraoral camera in teledentistry

**DOI:** 10.1038/s41405-023-00145-9

**Published:** 2023-04-29

**Authors:** Charnchai Santipipat, Issarapong Kaewkamnerdpong, Nareudee Limpuangthip

**Affiliations:** 1 Sisaket Provincial Public Health Office, Sisaket, Thailand; 2 Department of Community Dentistry, Faculty of Dentistry, Chulalongkorn University, Bangkok, Thailand; 3 Department of Prosthodontics, Faculty of Dentistry, Chulalongkorn University, Bangkok, Thailand

**Keywords:** Dental epidemiology, Gum disease

## Abstract

**Objectives:**

To facilitate dental disease screening program in prisoners by testing the diagnostic accuracy of teledentistry examination in comparison to direct oral examination by a dentist.

**Materials and methods:**

This crossover study comprised three phases. Phase I, prisoner health volunteers (PHVs) enrolled teledentistry training for an intraoral camera (IOC) use. Phase II, the PHV used IOC for examining dental diseases of prisoners who reported dental-related problems, and captured symptomatic areas. The PHV and dentist independently determined tentative dental treatment need, comprising dental fillings, scaling, extraction, and surgical removal of impacted tooth. Phase III, another dentist performed direct oral examination of the prisoners who reported problems in phase II and determined dental treatment needs. Sensitivity, specificity, positive predictive value (PPV), and negative predictive value (NPV) were calculated, using direct oral examination by dentist as a true positive.

**Results:**

Diagnostic accuracy was determined in 152 prisoners with 215 teeth. Sensitivity, specificity, PPV, and NPV of teledentistry and direct examination between two dentists were above 80%. The lowest sensitivity and specificity of teledentistry examination by the PHV were scaling and surgical removal.

**Conclusions:**

IOC use in teledentistry facilitates dentists in dental diseases screening for prisoners with acceptable diagnostic accuracy in identifying possible treatment needs. However, the imaging obtained from teledentistry is not adequate to accurately identify all dental treatment needs.

## Introduction

Teledentistry is the use of information and communication technologies as a component of oral health care to support oral health care delivery, consultation, referral, and patient-health professional communication and knowledge sharing [1]. The use of teledentistry increases oral health service accessibility, decreases the human resource and financial burden, and improves oral health of people [2]. Teledentistry reduces inequity in oral health care by increasing oral healthcare accessibility during unfavorable circumstances, such as the COVID-19 pandemic, and some restricted locations, such as remote areas, long-term care facilities, and prisons [3–5].

Due to crowded prisons circumstances with limited healthcare personnel, oral health problems have been reported among prisoners, including dental caries, periodontal disease, and tooth loss with dental prostheses treatment needs [6, 7]. The negative impact of poor oral health on the quality of life of the prisoners has been reported [8]. However, in Thailand, there are barriers to prisoners accessing oral healthcare because bringing them out of the prison is a cumbersome process, and the number of accompanying prison officers is limited [9].

Since 2019, the Ministry of Public Health of Thailand has established and conducted a health care project to address the oral health problems among prisoners nationwide, called the ‘Good Health Good Heart’ project. The aim of this project is to elevate the health and welfare of prisoners to be on par with those of the public [10]. Healthcare for prisoners focuses on improving the health service system, disease prevention, and health promotion, as well as a providing psychological health and oral healthcare services. The two oral health strategies are; an oral health-screening program for the prisoners that is performed by dental personnel and prisoner health volunteers (PHVs), and providing oral healthcare services, including oral disease prevention, oral health promotion, and dental treatment [10]. The PHV is responsible for providing health promotion, disease prevention, basic health support, as well as disease surveillance and screening for the prisoners and other prison staff [11, 12]. In Thailand, dental personnel and equipment are not generally available in prisons except for the Correctional Hospital located in Bangkok, which is under the jurisdiction of the Department of Corrections, Ministry of Justice. This hospital has dental professionals who can provide treatment and accept referrals from prisons in Bangkok. However, due to a shortage of dental professionals in other provincial prisons, the responsibility for providing dental treatment services falls to the public hospitals closest to the prison and the provincial public health office, which are under the jurisdiction of the Ministry of Public Health [12]. Additionally, a dental team administers a one-day mobile dental service in the prison at least four times a year, which primarily focuses on tooth extractions.

In addition to the obstacles of imprisonment, since the COVID-19 pandemic began, healthcare personnel have experienced more difficulty in gaining access to the prisons due to rigorous disease control measures. Adopting the use of teledentistry might increase oral healthcare accessibility, and improve the prisoners’ oral health and quality of life. Therefore, the objective of this study was to facilitate a teledentistry dental disease screening program in prisoners by testing the diagnostic accuracy of teledentistry examination in comparison to direct oral examination by a dentist.

## Materials and methods

The present study was a crossover design conducted in Sisaket Provincial prison and a primary healthcare setting in Sisaket province, Thailand. The male-wing Sisaket Provincial prison was chosen for data collection because the number of prisoners and PHVs was higher than those of female wing. Thus, male prisoners and PHVs were eligible for study participation. The exclusion criteria were the prisoners with a history of violent behavior and those not willing to have an oral examination by the healthcare personnel. The study protocol was approved by the Human Research Ethics Committee, Sisaket provincial public health office, Sisaket province (Ethical number: SPPH 2022-045), and performed in accordance with the Declaration of Helsinki Ethical Principles for Medical Research Involving Human Subjects. All participants agreed to and signed an informed consent prior to study participation.

The dental disease screening program consisted of three phases:

Phase I: Teledentistry training program for the PHVs

Phase II: Dental disease screening using teledentistry

Phase III: Oral examination by the dentist and dental treatment need evaluation

**Fig. 1:**
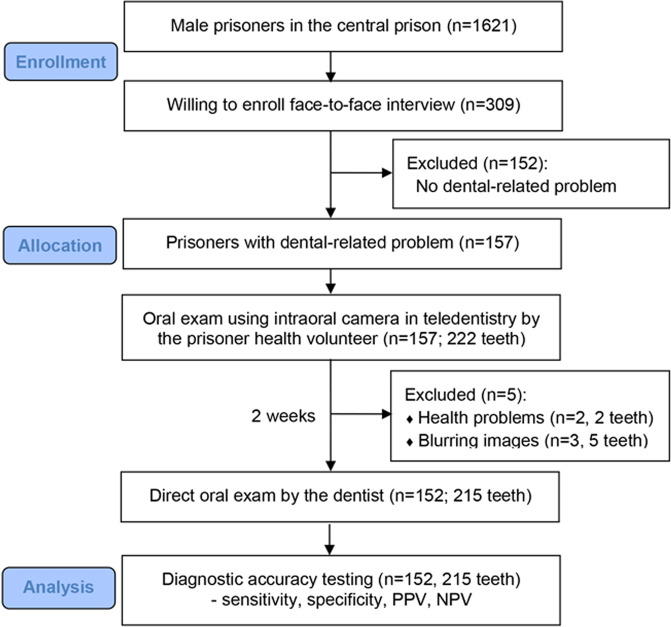
Flow diagram of the participants’ inclusion.

The phase II, teledentistry screening program, was conducted during August and September 2022. Phase I and phase III was carried out 2 weeks before and after phase II, respectively. The flow diagram for the participants’ inclusion in phase II and III is shown in Fig. 1.

In Thailand, the PHVs were volunteered prisoners with good time allowance who were selected by the prison chief. They must be illiterate and graduate at least elementary level. The PHVs had to complete the 25-hour health training program, which included oral health knowledge and practice [11]. The PHVs are provided basic knowledge in caries risk assessment, oral hygiene practice, food and nutrition, screening of oral diseases and their risk factors. They have to be able to identify possible treatment needs, comprising dental fillings, root canal treatment, dental scaling, simple tooth extraction, and surgical removal of an impacted tooth.

Dental caries detection and assessment followed the International Caries Detection and Assessment System (ICDAS) [13], of which the score ranged from 0 to 6. Teeth with the ICDAS score 0 to 2 indicated no treatment needed. Teeth with the ICDAS score 3 to 5 and patients’ complaint about having tooth sensitive, toothache, tooth cavity, or black discoloration indicated dental filling need. Teeth with the ICDAS score 6 and patients’ complaint about having a tooth cavity or broken tooth with severe or sudden toothache indicated dental filling, root canal treatment, or tooth extraction. However, since an x-ray machine is not available in the prison, it is not possible to use radiographic examination to differentiate whether the teeth need dental filling or root canal treatment. For the prisoners, tooth extraction would be the only a treatment option for the teeth with ICDAS 6 rather than root canal treatment. This is because the teeth with extensive cavitation may need root canal treatment, and endodontically-treated teeth require a prosthesis, such as a post, core, and crown, which is not a feasible treatment option for the prisoners due to its expense and time consumption.

The criteria for dental scaling needed was adopted from the simplified oral hygiene index [14]. The symptomatic non-cavitated tooth with supragingival calculus covering more than one third, or the presence of subgingival calculus around cervical third of the tooth indicated dental scaling needed. An impacted tooth with patients complained about having a toothache or tooth cavity, typically a third molar tooth that could not erupt vertically and might be covered by soft tissue, was an indication for surgical removal. According to evidence-based practice developed by the American Association of Oral and Maxillofacial Surgeons, impacted third molars should be removed or regularly monitored for the development of disease or pathology, even if they are currently asymptomatic [15]. However, in this study, only prisoners with dental problems were included, and all impacted third molars were symptomatic, indicating a need for removal.

### Phase I: Teledentistry training program for the PHVs

**Fig. 2:**
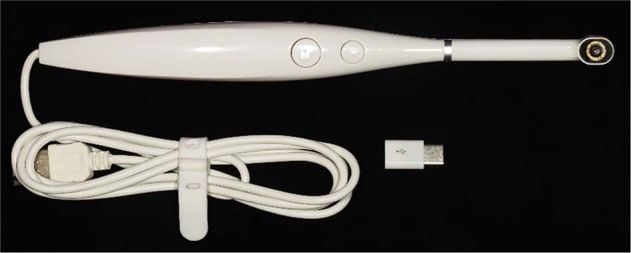
The intraoral camera used in teledentistry.

Eight out of 45 male PHVs in the Sisaket Provincial prison volunteered to enroll in the 1-day, 3-hour ‘Teledentistry Training Program for PHVs’. The PHVs were trained for using an intraoral camera or IOC (Portable Waterproof HD Video USB Stomatoscope Mouth Mirror, China), which is an imaging tool used in this teledentistry program (Fig. 2) [16]. The IOC can capture a picture with a resolution of 2 million pixels. A light source comprising 6 light emitting diode bulbs provides 3 levels of brightness with a visual acuity distance of 2–4 cm. The camera can connect with and transfer data to a computer or Android smartphone via a universal serial bus (USB 2.0) or microcontroller (microC) that displays an image with a resolution of 1600 × 1200 and 1280 × 720 pixels, respectively. While it is possible to transfer images from the IOC using Wi-Fi or cellular connection, these systems are not available in the prison. Therefore, in this study, images from the IOC were stored and synced to the VideoLAN Client (VLC) media player program, which is a free application available for Windows, iOS, and Android systems. To use the IOC in the prison, the VLC program was opened on a computer. After taking an intraoral photograph, the image was stored in a folder built specifically for each prisoner in the VLC program. Only the assigned nurse was authorized to transfer the images via a Google link or Excel sheet and send them to the dentist in the primary care cluster.

The PHVs were calibrated with a dentist using 30 intraoral photographs that comprised dental caries and periodontal diseases, and determined the possible treatment options. The PHV had to achieve at least a 0.80 Kappa value in agreeing with the dentist’s diagnosis prior to being permitted to perform data collection in phase II. There was only one computer that could be connected to an IOC in Sisaket Privincial prison office. Therefore, one trained male PHV was randomly selected by random number to participate the phase II teledentistry program.

### Phase II: Oral health and treatment need screening using teledentistry

From a total of 1621 male prisoners, 309 of them were willing to participate the dental disease screening by using the teledentistry. The PHV performed face-to-face interviews with the prisoners. The prisoner’s information was recorded, comprising their demographic information, systemic diseases, and a dental-related problem. The 157 prisoners with a dental-related problem pointed out the problematic areas in their oral cavity, and the PHV used an IOC to capture the potential symptomatic areas. The camera tip was covered with plastic wrap, and after each patient examination, the wrap was discarded, and the IOC tip was disinfected by wiping it with a towelette impregnated with a disinfection solution (CaviWipes^TM^) for 3 min, wiping with a 70% alcohol solution, and the tip was covered with a new plastic wrap. The prisoner’s information and intraoral photographs were recorded on a Google form and its link was connected with the dentist in the primary care cluster.

After the teledentistry screening program, the PHV and a dentist independently determined the tentative dental treatment needs for each individual tooth based on the intraoral photographs and the patients’ symptoms. The treatment needs consisted of a dental filling, dental scaling, tooth extraction, and the surgical removal of an impacted tooth. The dentist also provided the initial treatment plan for each prisoner for subsequent dental treatment appointments.

### Phase III: Direct oral examination by the dentist and dental treatment need evaluation

Another dentist who did not participate in phase II performed direct oral examinations of the prisoners who complained about having a dental-related problem. The dental treatment needs for each tooth were recorded, comprising a dental filling, dental scaling, tooth extraction, and the surgical removal of an impacted tooth. On the examination date, some prisoners underwent a simple tooth extraction in a mobile dental unit in the prison. The other required dental treatments were provided later at the primary care clusters.

### Power of the study calculation

The power analysis of sample size was calculated using G*Power version 3.1.9.4 program based on a two-sided 95% confidence interval for a single proportion using the exact-test family. Based on an effect size of 0.17 and a sample size of 157 with a prevalence of reported a dental-related problem (observed proportion) of 0.50, a 98.6% power was achieved.

### Data analysis

The SPSS program version 28.0 was used for data analysis. Descriptive statistics was used to demonstrate the mean (±standard deviation, S.D.) and frequency (percentage, %). For the diagnostic testing, four values were calculated for teledentistry examination by the PHV and dentist: sensitivity, specificity, positive predictive value (PPV), and negative predictive value (NPV). The true positive cases were identified based on a direct oral examination by the dentist in the phase III. The treatment needs determined by the PHV using teledentistry were compared with those of the dentist using teledentistry and the direct oral examination by the other dentist.

## Results

**Fig. 3:**
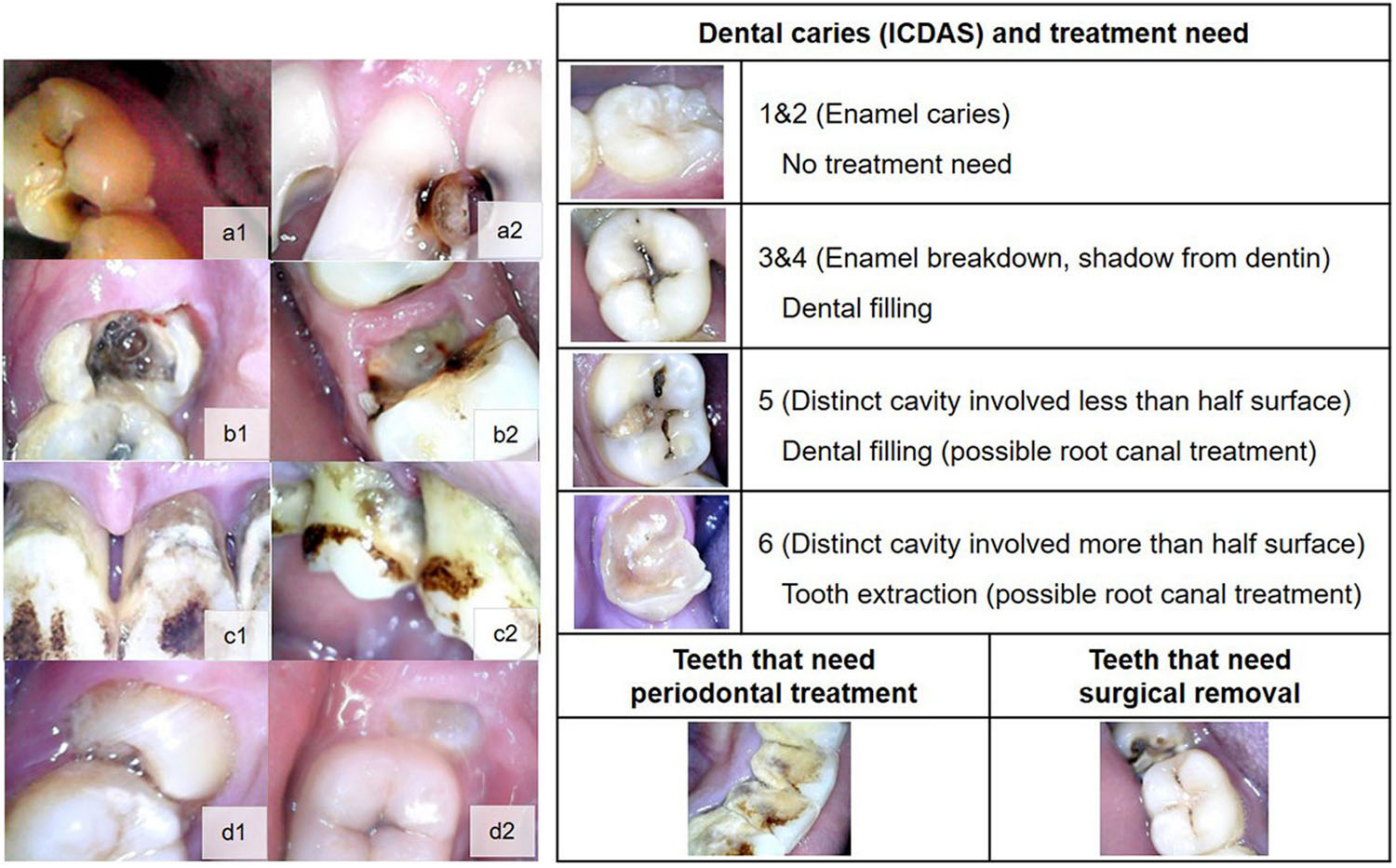
Intraoral photograph taken by intraoral camera. The photographs indicated a treatment need for dental filling (a1–a2), simple extraction (b1–b2), dental scaling (c1–c2), and surgical removal of impacted tooth (d1–d2), presented alongside the corresponding ICDAS table and the recommended dental treatment need. RCT, root canal treatment.

In phase II, 309 male prisoners participated dental disease screening program in teledentistry, and 157 of them who reported dental-related problems were eligible for further direct oral examination by a dentist. However, 5 prisoners were dropped out in phase III; 2 of them were unable to participate due to health problems, and 3 prisoners with 5 intraoral photographs were excluded due to the blurring images. Therefore, the study participants in phase III comprised 152 male prisoners with 215 symptomatic teeth. The participants’ mean age was 33.6 (±9.1) years old (range 19–67 years old). Table 1 demonstrates the baseline characteristics of the participants. The major chief complaints were toothache, tooth cavity, and a broken tooth. Representative intraoral photograph of the teeth taken by the IOC that needed dental filling, dental scaling, simple extraction, and surgical removal are seen in Fig. 3.

**Table 1:** Characteristics of the participants.

Variables	*N* (total = 152)	%
Age (years): mean (±s.d.)	33.6 (±9.1)	
Patient-reported symptoms (chief complaint):		
Toothache	58	38.2
Tooth cavity	42	27.6
Broken tooth	35	23.0
Tooth mobility	13	8.5
Tooth sensitive	3	2.0
Do not know	1	0.7
Tooth sites with the problem (total = 215 teeth):		
Maxillary anterior	26	12.1
Maxillary posterior	72	33.5
Mandibular anterior	6	2.8
Mandibular posterior	111	51.6

Table 2 showed the number of teeth that needed different dental treatment as diagnosed by teledentistry and direct oral examinations. The sensitivity, specificity, PPV and NPV of the teledentistry and direct oral examinations are illustrated in Table 3. The sensitivity and specificity of the teledentistry examinations by the two dentists were above 90%, and the PPV and NPV ranged from 81.8%–100.0% (Supplementary Tables). In addition, the sensitivity and specificity of the teledentistry examinations by the PHV ranged from 33.3–99.0% with the lowest percentage found for dental scaling and surgical tooth removal. The PPV for requiring a dental filling, dental scaling, and surgical tooth removal ranged from 40–60%, whereas the lowest NPV was seen for simple tooth extractions.

**Table 2:** The number of teeth that need different dental treatments determined by teledentistry and oral examinations (*N* = 215).

Dental treatment need	Teledentistry examination		Direct oral examination by 2nd dentist
	by PHV	by 1st dentist	
Dental filling	78	38	35
Dental scaling	5	9	6
Simple extraction	127	159	165
Surgical removal of impacted tooth	5	9	9

**Table 3:** Diagnostic accuracy testing between the teledentistry and direct oral examinations.

Teledentistry examination	Sensitivity (%)	Specificity (%)	PPV (%)	NPV (%)
PHV: Dental filling	97.1	81.1	50.0	99.3
Dental scaling	33.3	98.5	40.0	98.1
Simple extraction	75.8	86.0	94.7	51.8
Surgical removal of impacted tooth	33.3	99.0	60.0	97.1
Dentist: Dental filling	97.1	97.2	89.5	99.4
Dental scaling	100.0	100.0	100.0	100.0
Simple extraction	93.9	98.0	99.4	83.1
Surgical removal of impacted tooth	100.0	98.6	81.8	100.0

## Discussion

Our findings demonstrated high sensitivity, specificity, PPV and NPV in determining the dental treatment needs between two dentists using teledentistry and direct oral examination. However, these values were relatively low when comparing the teledentistry examination by the PHV and direct oral examination by the dentist. The first study using oral teleconsultation in a prison was performed by Giraudeau et al. [5] and several studies have utilized the IOC in teletedentistry for oral examination of prisoners [5, 17, 18]. However, to our knowledge, our study is the first to assess the validity, regarding sensitivity and specificity, of using teledentistry for dental disease screening in comparison to the direct oral examination by a dentist.

Several imaging tools have been used in teledentistry, including an intraoral scanner [19], digital cameras, smartphone cameras, and an IOC [20, 21]. In the present teledentistry program, the IOC was used due to its low cost, being convenient to transport, and easy to maintain. The IOC has been used for patient-dentist communication [5, 22], oral examination [17, 18], screening oral cancer lesions [23], and identifying dental diseases and soft tissue conditions [21, 24]. Previous studies have found that the IOC is a valid and reliable tool for screening oral diseases, such as dental caries [21] and oral cancer [23], compared with a direct oral examination. We found that the IOC used in the present teledentistry demonstrated acceptable diagnostic accuracy in determining the prisoners’ dental treatment needs compared with a direct oral examination.

Although the teledentistry and direct oral examinations by the dentists were highly consistent, some disagreements existed between the teledentistry examination findings by the PHV and the direct oral examination by the dentist. Approximately 50% PPV for a dental filling and NPV for a simple tooth extraction were demonstrated when comparing the teledentistry examination by the PHV and the direct oral examination by the dentist. This finding might be because the PHV underestimated the treatment needs for dental pulp lesions. In some teeth with extensive dental caries, the PHV evaluated them as requiring a dental filling, however, the dentist determined that they required simple tooth extraction. Clinically, some extensive dental caries teeth may undergo root canal treatment, and prosthetic rehabilitation, however for the prisoners, this treatment option is not available because several clinical appointments outside the prison are required. Therefore, in addition to the IOC resolution in providing a distinct intraoral photograph, the treatment need decision depends on the clinical experience of the healthcare personnel to decide on the optimal treatment option in a specific situation. Our results suggest that the PHVs may need further training and clinical experience to make a more accurate treatment decision.

The 33.3% sensitivity of the teledentistry examination of the dental scaling need indicated 66.6% false negative results, and its PPV was only 40.0%. This relatively high amount of inconsistency was because gingivitis is not a clearly observed lesion, and gingival bleeding or inflammation cannot be clearly identified from only an intraoral photograph. The PHV, therefore, made their treatment need decision based on the patient’s complaint of dental pain and misjudged them as a dental filling need. Therefore, in case where the prisoner has a dental compliant but demonstrates normal tooth structure, additional information should be included, such as bleeding on probing and calculus formation. A real-time teledentistry examination between the PHV onsite and the dentist online might be used to increase the diagnostic accuracy of the dental treatment needs.

There were some inconsistencies between the direct oral and teledentistry examinations by the two dentists, particularly about whether a simple extraction or surgical removal was needed. Some retained roots were determined as having a simple extraction need based on the teledentistry examination, however, the direct oral examination indicated that they required surgical removal. Some restorable teeth were determined as having for a dental filling need, however, the direct oral examination found high tooth mobility, which requires extraction. These findings indicate that an intraoral photograph might not be adequate for making an accurate diagnosis because it provides only a two-dimensional image. In contrast, direct oral examination provides a three-dimensional view and allows for detecting tooth movement. Moreover, in opposing to a direct oral examination, teledentistry examination cannot accurately determine pulpal health that would dictate dental treatment need because diagnosis of endodontic disease requires thermal and electric pulp sensibility tests [25].

There are some limitations in the present study. Only one PHV was included so it is unclear whether the same accuracy level would be achieved if using multiple PHVs. Only the symptomatic areas were investigated which limits the generalizability and validity of the results as it decreases the chance of false positives. Also, recall bias from the prisoner-report symptoms or problems could occur, resulting in underestimation of the disease prevalence. Some information, including radiographic examination and probing depth, was not collected to provide a definitive diagnosis and treatment plan. Teledentistry examinations cannot accurately identify the need for periodontal treatment because the IOC and imaging are unlikely to capture subgingival calculus deposits. In addition, they cannot measure bleeding on probing, periodontal pocket depth, or furcation involvement, which are included in the basic periodontal examination, a conventional screening tool [26]. Furthermore, the imaging cannot provide information that can be obtained from tactile evaluation, such as active or arrested dental caries.

There are several advantages of establishing a dental disease screening program for prisoners using teledentistry. The dental disease screening can be performed by other trained health personnel without requiring direct examination by a dentist. This reduces the time and the number of human resources required, including dentists, dental assistants, and prisoner officers, as well as the number of dental visits. Integrating teledentistry screening into admission protocols would be beneficial to promptly assess treatment needs in prisons with high turnover rates and limited dental personnel to perform regular dental check-ups. Teledentistry enables the prioritization of dental treatment needs, facilitates the organization of face-to-face appointments, and allows for preparation of required equipment and mobile dental units. Furthermore, although teledentistry cannot completely replace traditional face-to-face oral health care and dental treatment, it can be used for making a tentative diagnosis and treatment planning.

For further studies, using multiple PHVs would be further required when the oral health screening is conducted in a larger sample size. Other periodontal health indicators, including probing depth and intraoral radiographs might be collected to provide a more accurate diagnosis and treatment need determination. Using the IOC to record a video for additional information, such as tooth mobility and gingival bleeding may be required to increase diagnostic accuracy of dental treatment need.

## Conclusion

Teledentistry facilitates dentists in conducting dental diseases screening program for prisoners. Using the IOC, the dentists achieved acceptable diagnostic accuracy in identifying the possible dental treatment needs compared with direct oral examination. However, the imaging obtained from teledentistry is not adequate to accurately identify all dental treatment needs due to limitations in making a definite diagnosis of pulp and periodontal status.

## Supplementary information

Supplementary Information

## Data Availability

The raw data of this study will be provided upon request to the corresponding author.
