# Supplementary material for: Facilitating dental disease screening program in prisoners using an intraoral camera in teledentistry
Source: BDJ Open. 2023 Apr 29;9:18. doi: 10.1038/s41405-023-00145-9 (PMC10148622; doi:10.1038/s41405-023-00145-9)
Supplement: Supplementary file 1 — Supplementary Information [file 41405_2023_145_MOESM1_ESM.pdf]

Supplementary Tables

Supplementary Table 1. Diagnostic testing for dental filling (N=215 teeth)

Teledentistry examination by PHV	Oral examination by dentist (Filling)		Total
	Yes	No	
Yes	34	34	68
No	1	146	147
Total	35	180	215

Teledentistry examination by dentist	Oral examination by dentist (Filling)		Total
	Yes	No	
Yes	34	4	38
No	1	176	177
Total	35	181	215

Supplementary Table 2. Diagnostic testing for dental scaling (N=215 teeth)

Teledentistry examination by PHV	Oral examination by dentist (Dental scaling)		Total
	Yes	No	
Yes	2	3	5
No	4	206	210
Total	6	209	215

Teledentistry examination by dentist	Oral examination by dentist (Dental scaling)		Total
	Yes	No	
Yes	6	0	6
No	0	209	209
Total	6	209	215

Supplementary Table 3. Diagnostic testing for simple extraction (N=215 teeth)

Teledentistry examination by PHV	Oral examination by dentist (Simple extraction)		Total
	Yes	No	
Yes	125	7	132
No	40	43	83
Total	165	50	215

Teledentistry examination by dentist	Oral examination by dentist (Simple extraction)		Total
	Yes	No	
Yes	155	1	156
No	10	49	59
Total	165	50	215

Supplementary Table 4. Diagnostic testing for surgical removal (N=215 teeth)

Teledentistry examination by PHV	Oral examination by dentist (Surgical removal)		Total
	Yes	No	
Yes	3	2	5
No	6	204	210
Total	9	206	215

Teledentistry examination by dentist	Oral examination by dentist (Surgical removal)		Total
	Yes	No	
Yes	9	2	11
No	0	204	204
Total	9	207	215
